# Cervical Cancer Screening in Older Women: New Evidence and Knowledge Gaps

**DOI:** 10.1371/journal.pmed.1001586

**Published:** 2014-01-14

**Authors:** Anne F. Rositch, Michelle I. Silver, Patti E. Gravitt

**Affiliations:** 1 Department of Epidemiology and Public Health, University of Maryland School of Medicine, Baltimore, Maryland, United States of America; 2 Department of Epidemiology, Johns Hopkins Bloomberg School of Public Health, Baltimore, Maryland, United States of America; 3 Department of Pathology, University of New Mexico Health Sciences Center, Albuquerque, New Mexico, United States of America

## Abstract

Anne Rositch and colleagues discuss the study by Peter Sasieni and colleagues on cervical cancer screening in older women and describe the further information needed to help inform decisions about whether to extend screening programs beyond 65 years for women with adequate negative screening.

*Please see later in the article for the Editors' Summary*

**Linked Research Article**

This Perspective discusses the following new study published in *PLOS Medicine*:

Castañón A, Landy R, Cuzick J, Sasieni P (2014) Cervical Screening at Age 50–64 Years and the Risk of Cervical Cancer at Age 65 Years and Older: Population-Based Case Control Study. PLoS Med 11(1): e1001585. doi:10.1371/journal.pmed.1001585

Peter Sasieni and colleagues use a population-based case control study to assess the risk of cervical cancer in screened women aged over 65 years to help inform policy on the upper age of cervical cancer screening.

Recently, an expert panel was convened on behalf of the American Cancer Society (ACS), the American Society for Colposcopy and Cervical Pathology (ASCCP), and the American Society for Clinical Pathology (ASCP) to review the evidence for the age to stop cervical cancer screening. The paucity of data needed to guide this recommendation was noted by a call for prospective studies in older women as a key research priority. In this week's issue of *PLOS Medicine*, Sasieni and colleagues, contribute important new data on risks of cervical cancer in older women with different screening histories [1].

## Current Recommendations and Controversies in Cervical Cancer Screening in Older Women

The consensus guidelines recommend cessation of routine cervical cancer screening at age 65 for women with a history of adequate negative screening, defined as three consecutive negative cytology results, or two consecutive negative co-tests in the past 10 years [2]. Recently, this recommendation has been challenged [3], citing insufficient consideration of studies that demonstrate the protective effects of screening in older women, including several case-control studies and audits of national cervical cancer screening programs [4]–[8]. All studies confirm the higher risk of cervical cancer in women with recent abnormal screening. Thus, when considering the adequacy of the current guidelines, the primary question is not one of overall screening effectiveness, but rather, at what age is there a sufficiently low risk in older women with previous normal screening tests to safely exit them from the screening program. Unfortunately, there have been few reports that provide estimates of risk of invasive cervical cancer (ICC) following negative screening tests in older women to adequately inform this decision. In one recent study from the Netherlands, the 10-year cumulative risk of ICC following three consecutive negative screening tests in women with no history of cervical intraepithelial neoplasia (CIN) or cytological abnormalities was similar in women who were 30–44 and 45–54 years old at the time of their last negative screen [9]. These data support continued screening up to at least age 55, but do not directly inform risk at older ages. A small study in the United States showed that the incidence of ICC in women age 55–79 was reduced in the first few years following the last negative screening test, but returned to rates observed among unscreened women within 5–7 years [8].

## New Evidence for Effectiveness of Cervical Cancer Screening after Age 65

Using a case-control study design, Sasieni and colleagues found that women in the UK aged 50–64 years with an adequate negative screening history (defined as at least three tests at age 50–64, the last three of which were negative with at least one at 60–64, and no high-grade [HSIL] or worse cytology since age 50) had 80% less risk of cervical cancer diagnosis after age 65 (4/100,000) compared to women who were not screened between 50 and 64 (24.5/100,000) [1]. The low risk among women with an adequate negative screening history at age 65 supports the principle tenet that risk following a series of normal cytology screening tests is significantly lower than the risk associated with a recent abnormal screening test, justifying continued screening in the latter group. However, similar to the US study, the protective effect afforded by adequate negative screening waned significantly with time.

How long, then, should screening continue in women with adequate negative history? In the study by Sasieni and colleagues, Table 8 provides a particularly useful analysis to address this issue [1]. The authors estimate that stopping screening at age 55 would result in nearly twice the number of cancers compared to exiting at age 65, which itself would result in twice the number of cancers compared to exiting screening at age 75. These data suggest a lifelong protective benefit of screening even in women with an adequate negative history. Thus, the decision of whether to stop screening earlier or later than age 65 may rest heavily on the perceived harms of screening and whether the harm to benefit ratio increases significantly with age [10]. Using number of colposcopies per life-year as an estimate of harm in screening, models that evaluated extending screening to age 75 using 5-year interval screens resulted in only two additional colposcopies that translated to 20 additional colposcopies per life-year [11]. Given the projected 2-fold increase in the number of cancer cases averted by extending screening to age 75 reported by Sasieni and colleagues [1], it appears that alternative ages to exiting screening with acceptable harm to benefit ratios are available.

## Evidence to Support Reconsideration of Recommendations for Cessation of Screening

In addition to the data presented above, several other issues may factor considerably into the evaluation of the benefit of extending routine screening beyond age 65 for women with adequate negative screening. First is whether the cumulative risk in these women is sufficiently low to warrant cessation of screening in their remaining years. A key evidence gap to inform this decision is the lack of qualitative data to determine the perceived balance in benefits and risks associated with screening in previously well-screened older women. The reproductive harms associated with screening are no longer relevant in older women, and it is unclear whether the discomfort and false positive test results are significantly different in women 55–65 compared to 65–75 years and whether these risks outweigh the perceived benefits. Second, because modeled estimates rather than empirical data were used for formal evaluation of the relative harm to benefit of alternative ages to exit screening, it is important to critically evaluate the validity of the key assumptions and parameters driving these models. One must question whether updates are needed to better account for: (1) the high prevalence of hysterectomy in estimating population-level rates of cancer at older ages; (2) the growing evidence supporting HPV latency and reactivation as possible explanations for “new” HPV detection at older ages [12],[13]; (3) the higher lifetime exposure to HPV in the birth cohorts nearing the age to exit screening who had sexual debut during the sexual revolution [14]; and (4) age-specific differences in the sensitivity and specificity of screening [15]. Incorporating the new data on older women, such as those referenced above and presented by Sasieni and colleagues [1], into the evaluation of whether to extend screening beyond age 65 for women with adequate negative screening will provide much needed insight into whether current guidelines are sufficient for the population now and in the future.
